# Senataxin and R-loops homeostasis: multifaced implications in carcinogenesis

**DOI:** 10.1038/s41420-023-01441-x

**Published:** 2023-05-05

**Authors:** Veronica Gatti, Sara De Domenico, Gerry Melino, Angelo Peschiaroli

**Affiliations:** 1 National Research Council of Italy, Institute of Translational Pharmacology, Rome, Italy; 2 Department of Experimental Medicine, TOR, University of Rome Tor Vergata, Rome, Italy

**Keywords:** Cancer genetics, Tumour-suppressor proteins

## Abstract

R-loops are inherent byproducts of transcription consisting of an RNA:DNA hybrid and a displaced single-stranded DNA. These structures are of key importance in controlling numerous physiological processes and their homeostasis is tightly controlled by the activities of several enzymes deputed to process R-loops and prevent their unproper accumulation. Senataxin (SETX) is an RNA/DNA helicase which catalyzes the unwinding of RNA:DNA hybrid portion of the R-loops, promoting thus their resolution. The key importance of SETX in R-loops homeostasis and its relevance with pathophysiological events is highlighted by the evidence that gain or loss of function SETX mutations underlie the pathogenesis of two distinct neurological disorders. Here, we aim to describe the potential impact of SETX on tumor onset and progression, trying to emphasize how dysregulation of this enzyme observed in human tumors might impact tumorigenesis. To this aim, we will describe the functional relevance of SETX in regulating gene expression, genome integrity, and inflammation response and discuss how cancer-associated SETX mutations might affect these pathways, contributing thus to tumor development.

## Facts

- Senataxin (SETX) is an RNA/DNA helicase which catalyzes the resolution of R- loops, inherent byproducts of transcription consisting of an RNA:DNA hybrid and a displaced single-stranded DNA.
- Gain or loss of function mutations of SETX gene underlies the pathogenesis of two distinct neurological disorders: AOA2 (ataxia with oculomotor apraxia - type 2) and ALS4 (juvenile Amyotrophic Lateral Sclerosis), respectively.
- SETX physiological functions ranging from gene expression regulation to genome integrity maintenance and inflammation response.
- SETX dysregulation has been observed in human tumors and several evidence pointed towards a tumor suppressive role of this helicase.

## Open Questions

- Are SETX defects directly involved in tumor onset and progression?
- Which specific SETX functions underlie its potential tumor suppressive role?
- Is it possible that SETX gain of function alterations might drive tumor development, similar to what is observed in neurological context?

## Introduction

Cells have evolved an intricate network of signaling activated in response to DNA damage that ensures that DNA lesions might not be converted into heritable mutations. Paradoxically, the major sources of mutagenic events are represented by physiological processes involving DNA and RNA metabolism. During DNA replication or gene transcription DNA undergoes a series of changes which may generate structural intermediates which can facilitate DNA mutations. An example of such structural intermediates are R- loops, RNA:DNA hybrids generated during transcription when the non-coding DNA strand is displaced as the transcribed DNA strand forms a hybrid with the nascent pre-mRNA [1, 2]. These inherent byproducts of transcription are of key importance in controlling physiological processes and are present in the genomes of most known organisms occupying a significant portion of the genomes [3, 4]. As a general point, R-loops form prevalently at highly expressed *loci*, including either coding or noncoding sequences, and their accumulation/stabilization is promoted at specific DNA regions, typically enriched with purine-rich sequences, or during replication-transcription collisions [5].

R-loops formation can impact numerous biological processes, such as antibodies diversification in B cells, DNA damage response and gene expression at multiple levels, including regulation of the chromatin architecture at the promoter region, transcription elongation, and termination [6–10]. In human cells, genes containing CpG island promoters are characterized by R-loops which are formed downstream of the transcription start site until the first exon-intron junction [11–13]. R-loop formation at these gene promoters prevents DNA methylation and promotes a transcription permissive chromatin status, which ultimately leads to gene expression [14]. An additional genomic hot spot for R-loops formation is represented by the transcription gene terminators. On these genomic elements R-loops formation induces the pausing of RNA polymerase II facilitating an efficient transcription termination [15]. In addition, R-loops formation on gene terminators triggers the recruitment of the enzyme responsible for writing the repressive mark H3K9me2, an epigenetic feature of the terminator elements of certain highly expressed genes [16]. In addition to gene expression regulation multiple evidence indicate the central role of R-loops in modulating DNA repair and by doing this, maintaining genome integrity [17, 18].

Although physiologically relevant for many biological processes, the persistence of the R-loops might be deleterious for cell viability [19–21]. Therefore, R-loop homeostasis needs to be finely balanced and several enzymes are deputed to resolve R-loops and prevent their unproper accumulation. Generally, exon-intron sequences act as a barrier for R-loop spreading into genomes and splicing factors, such as SLU7 and SRSF1, prevent R-loop formation at the splicing sites allowing a proper RNA processing [22–24]. R-loop processing is catalyzed by two main class of enzymes: RNA-DNA helicase enzymes and nucleases. RNA-DNA helicase enzymes (BLOOM, DDX5, DDX19, DDX21, SETX, WERNER among others) catalyzed the unwinding of RNA:DNA hybrid portion of the R-loop promoting its resolution, while some RNA-DNA nucleases, such as RNAseH1 and RNAseH2, hydrolyze the RNA moiety in the RNA:DNA hybrids dissolving thus R-loops. Other endonucleases involved in the R-loop resolution are FEN1 that is able to cut both the displaced ssDNA and the RNA filament of an R-loop and XRN2 which degrades nascent RNA downstream the 3’terminal cleavage site. The pivotal importance of these R-loops resolving factors and their functional relevance in R-loops homeostasis is highlighted by the evidence that mutations or aberrant expression of some of these factors are functionally linked with the pathogenesis of diverse human diseases. The most compelling evidence of how dysfunctions of R-loops processing enzymes are linked to human health is represented by the RNA/DNA helicase Senataxin (SETX), whose mutations underlines the pathogenesis of two distinct neurological disorders [25, 26]. Many excellent reviews have extensively described SETX function inthe neurological context [27, 28]. Here, we aim to discuss the function of SETX in tumor-related pathways trying to emphasize how dysregulation of this enzyme might be beneficial or detrimental for tumor evolution. To this aim, in the next paragraphs we will describe the functional relevance of SETX in gene expression, genome integrity and inflammation response and discuss how cancer associated SETX mutations might contribute to the pathogenesis of human cancer.

## SETX functions: from gene expression to genome integrity and inflammation response

**Fig. 1:**
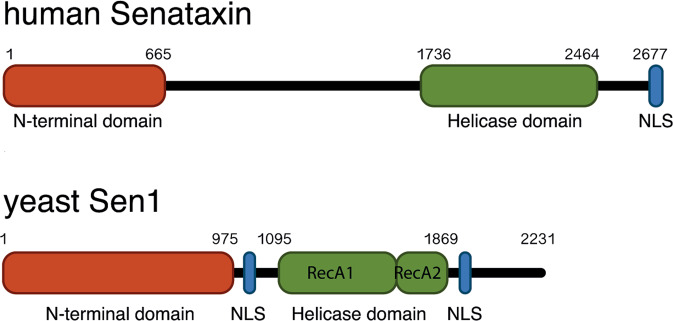
Schematic diagram of the functional domains of human senataxin and its yeast (*S. cerevisiae*) homolog senp1. N-terminal domains (red boxes), Helicase domains (green boxes) and Nuclear Localization Signals (NLS, blue boxes) are shown. Sen1 contains two RecA domains (RecA1 and RecA2) with the classical helicase motifs involved in nucleic acid binding and ATP hydrolysis. Numbers indicate amino acid residues.

SETX is one of the best-characterized R-loops resolving helicase enzyme. The human SETX gene is localized on chromosome 9 and includes 33 exons codifying a 302-kDa protein. SETX protein presents key protein domains: an N-terminal protein interaction domain, the helicase domain, and a C-terminal Nuclear Localization Signal (NLS) (Fig. 1). SETX is ubiquitously expressed and is localized mainly in the nucleus although cytoplasmatic localization has been reported [29, 30].

The scientific interest in this enzyme started in 2004 when two independent groups found that recessive and dominant mutations of SETX gene are responsible for the pathology of two distinct neurological disorders: AOA2 (ataxia with oculomotor apraxia - type 2) [25] and ALS4 (juvenile Amyotrophic Lateral Sclerosis) [26], respectively.

SETX mutations in AOA2 patients (approximately 120 mutations identified so far) are localized throughout the entire sequence and include missense, nonsense, splice site, frameshift, and insertion/deletions. ALS4 mutations are mainly localized in the N-terminal portion and in the helicase domain. The type of mutations associated with these neurological diseases supports the concept that SETX loss of function underlines the pathogenesis of AOA2, while SETX gain of function is responsible for ASL4 [25, 31–33].

The delineation of the physiological functions of human SETX has been markedly influenced by studies done on its yeast homolog, Sen1p which shares with the human SETX a conserved helicase domain, suggesting that both proteins may have similar functions. Indeed, SETX and Sen1p can resolve RNA Polymerase II-driven R-loops and direct the exosome to sites of transcription-replication collisions [34, 35]. Both SETX and Sen1p regulate gene expression and are key determinants of genome integrity. However, the SETX protein sequence is much longer respect to the Senp1 and the homology with Sen1p is restricted only to the helicase domain [36], suggesting that Senp1 and SETX might possess distinct functions and regulation. Indeed, while Senp1 primarily acts as regulator of the transcription termination of non-coding RNAs (ncRNAs) [37, 38], SETX is not able to control the transcriptional termination of ncRNAs [39], likely due to the absence of the human homolog of the RNA‐binding protein Nab3 necessary for the Senp1 function [40]. In the next paragraphs we will dissect the molecular functions of SETX. We will focus our discussion on three pathways in which the R-loops resolving activity of SETX is mainly involved: (i) regulation of gene expression, (ii) maintenance of genome integrity and (iii) inflammation.

### Role of SETX in gene expression regulation

**Fig. 2:**
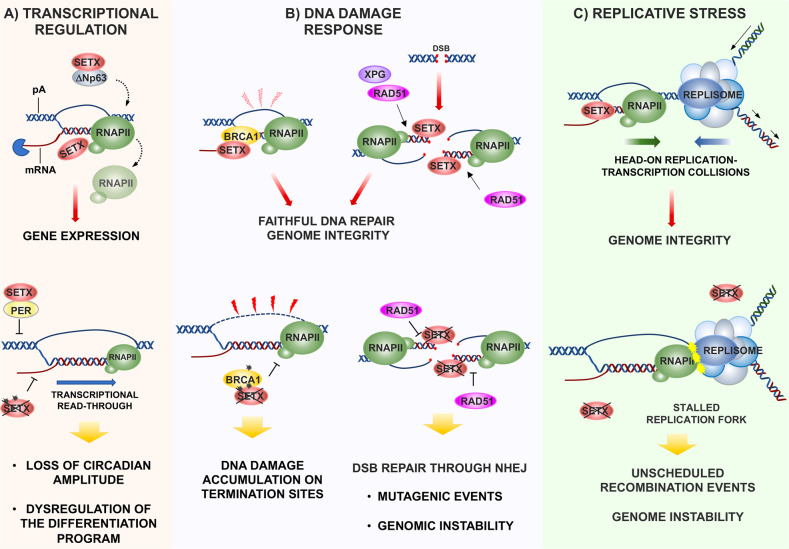
Schematic model of SETX function in controlling gene expression. **A)** SETX recruitment to transcription-termination sites promotes R-loops unwinding and proper transcriptional termination. SETX loss (black cross), mutation (black asterisks) or inhibition leads to transcriptional read-through. **B)** BRCA1-SETX interaction on termination sites, protects exposed ssDNA from DNA damage. Loss of this complex causes DNA damage accumulation on termination sites. SETX is also involved in DSB repair, and its loss is associated with genomic instability. **C)** SETX prevents the persistent accumulation of R-loops during head-on transcription replication conflicts favoring genome integrity and protecting from detrimental recombination events.

Similarly to its yeast homolog, SETX is involved in the regulation of gene expression. SETX interacts with RNA pol II and other RNA transcription and processing factors, such as poly(A)-binding proteins 1 and 2 (PABP1/2), hnRNPs, SAP155, SMN, SPT5, TAF4, KAP1/TRIM28, and TP63 [41–43]. These interactions underlie SETX function in the transcriptional regulation of a discrete number of housekeeping, tissue specific or stimulus-induced genes as reflected by the variable extent of transcriptional deregulation observed in SETX depleted cells [44, 45]. Since R-loops are transiently found at promoter regions, at transcription termination sites, as well as at highly transcribed gene bodies is not surprising that SETX is able to regulate gene expression at multiple levels. The most characterized and studied function of SETX on transcription regulation is related to its ability to resolve R-loops at the transcription termination sites, favoring thus transcription termination [15]. During transcriptional termination, transcribing RNA pol II slows down because of R-loos formed at termination elements located downstream of the poly(A) signal. SETX is able to unwind RNA/DNA hybrids formed behind the elongating RNA pol II at the termination sites allowing the release of the nascent RNA and promoting the degradation by the 5′–3′ exonuclease XRN2 (Fig. 2A). In addition, SETX depletion decreases RNA pol II density over the gene bodies, suggesting that SETX loss impacts both transcriptional termination and elongation. The relationship between 3′-end processing and transcriptional initiation is supported by the idea of coordinated recycling of transcription and processing factors from a gene terminator back to the promoter due to promoter-terminator “gene-loop” physical interactions.

The function of SETX to facilitate the transcriptional termination process is more relevant in highly expressed genes such as house-keeping genes. As line of principle, actively expressed genes should be more prone to undergo mutagenic events and therefore it is not surprising that R-loops processing at the termination sites and DNA damage response are functionally linked in order to limiting DNA lesions and preserve genome integrity. A relevant example of such functional interactions is represented by the ability of SETX to interact with BRCA1 [17], a tumor-suppressor gene mutated in breast, ovarian and other types of cancers [46–48]. BRCA1 and its related gene BRCA2 are crucial factors in the DNA damage response, as they are involved in the maturation of Rad51 nucleofilaments at initial stage of homologous recombination (HR) process [47]. Several evidence have functionally linked BRCA1 activity and R-loops homeostasis. BRCA1 is indeed recruited throughout hybrids at double strands breaks to promote HR and prevent R-loops accumulation at transcribed genes and at promoter-proximal pausing sites [49–52]. Mechanistically, BRCA1 interacts with SETX favoring R-loops processing at transcriptional terminator pause sites of actively expressed genes [17].

In addition to BRCA1, SETX might regulate transcription termination through its recruitment by other factors that might dictate gene specificity. In human keratinocytes the master epithelial transcription factor ΔNp63 is able to recruit SETX on the termination region of early epidermal differentiation genes favoring R-loops removal on those loci and allowing efficient transcriptional termination and gene expression [45] (Fig. 2A). Remarkably, SETX expression is significantly decreased, or its gene mutated in Squamous Cell Carcinomas of different origins, which are commonly characterized by dysregulation of the differentiation program [53].

SETX interacting partners can also inhibit its activity as it happens in the circadian clock oscillation where a molecular complex involving the repressor PER binds to SETX blocking subsequent processing by XRN2 [54] (Fig. 2A). This, in turn, causes an accumulation of RNA pol II near the 3′ termination sites and an increase in 3′-read-through at the circadian clock genes Per1 and Cry2. Remarkably, SETX loss causes a marked loss of circadian amplitude, suggesting an important role of SETX in the regulation of circadian clock. Many tumor types exhibit alterations of the circadian genes and impaired circadian rhythms may affect cancer initiation and progression [55]. For instance, colon carcinoma is one of the cancers whose pathogenesis is strictly associated with circadian disruption [56] and at the same time it is one of the human tumors exhibiting higher rate of SETX mutation (see below).

In addition to positively regulate gene expression, SETX may also act as transcriptional repressor. During meiosis, SETX-BRCA1 complex is recruited to XY sex chromosomes, where it mediates transcriptional sex chromosome inactivation [57]. Furthermore, during viral infection, SETX together with the transcriptional cofactor TAF4 promotes promoter-proximal termination of the transcription of antiviral genes, attenuating thus the antiviral response [43].

### SETX and genome integrity

Alterations of the DNA damage and DNA repair signaling are hallmarks of many, if not all, human cancers [48, 58–62]. Multiple line of evidence indicate that defects in R-loops homeostasis might interfere with the DNA damage response and impair DNA repair mechanisms [18, 63, 64]. One of the best examples of the functional relationship between SETX and DNA damage response is related to the functional interaction of SETX with BRCA1 (Fig. 2B). As discussed in the previous paragraph, BRCA1-SETX complex favors R-loops processing at the terminator pause sites of actively expressed genes [17]. Mutation or depletion of each of these two factors negatively influences transcription termination preventing efficient R-loops turnover on terminal elements leading to RNA pol II stalling and DNA damage accumulation on these loci. Notably, compared to sporadic breast cancers, BRCA1 tumors are significantly enriched for mutagenic events occurring in gene transcriptional termination regions. These observations clearly indicate that BRCA1-SETX complex is instrumental for preventing the R-loop-mediated DNA damage at the transcriptional termination sites, maintaining thus genome integrity and limiting cancer evolution.

Defects in R-loops homeostasis might interfere with the DNA damage response and impair DNA repair mechanisms, mainly when DNA lesions occurs at transcriptionally active regions. When double strand breaks (DSB) occur at transcribed loci, the transient formation of R-loops favors DNA repair and prevents illegitimate rejoining of broken ends [65]. R-loops trigger an XPG-dependent non-canonical mechanism of DSB resection, favoring HR over the error prone non-homologous end joining (NHEJ) mechanism [65]. The critical role of the R-loop resolving activity of SETX in modulating DNA repair signaling has been demonstrated by the analysis of SETX deficient cells, which display increased DSB-induced R-loops formation (Fig. 2B). At functional level, SETX loss prevents HR and increases mutagenic NHEJ by preventing the proper recruitment of RAD51 to the chromatin, impacting thus DSB repair fidelity [66]. At genomic level, these defects translate into the major propension of SETX deficient cells to undergo illegitimate rejoining of broken ends, which can lead to genomic translocations. Along the same line, another study showed that in response to DSB occurring at the transcribed loci SETX counteracts RNA:DNA hybrid accumulation, preventing thus large DNA deletions [67].

The critical role of SETX in preserving the genome integrity is also demonstrated by its ability to promote the removal of cytotoxic DNA interstrand crosslinks (ICLs) [68]. Generally, ICLs are repaired by a class of proteins which are inactivated in the cancer susceptibly syndrome Fanconi anemia (FA) [69]. Andrews et al., showed that SETX assists SAN1, a 5’ exonuclease acting independently of the FA pathway in response to ICLs, to dampen ICLs sensitivity.

In addition to impair DNA repair pathway, SETX loss might induce per se DNA damage by inducing transcription stress. Mouse and human studies have indeed demonstrated that SETX deficiency results into R-loop accumulation at promoter-proximal regions, which in turn promotes RNA Pol II pausing/stalling [70] (Fig. 2B). In the absence of SETX, R-loops near gene promoters are targeted and repaired by the repair mechanism involving the XPG/XPF nucleases, RAD52 and the transcription-coupled repair factor Cockayne syndrome B (CSB). These aberrant repair reactions induce accumulation of elevated levels of DNA damage and genomic instability.

Another example of the functional relationship between DNA damage and SETX has been recently reported by analyzing oocyte derived from SETX knock-out mice [71]. Subramanian and colleagues showed that Setx^+/−^ and Setx^−/−^ females are characterized by a decline of the ovarian activity, known as premature ovarian ageing (POA). In detail, SETX knock-out increases the DNA damage in ovarian follicles and reduces the ovarian follicular reserve. This phenotype does not seem to be related to defects of the DNA repair pathway but rather to the acquisition of de novo DNA damage, likely due to an increase of the oxidative stress. Intriguingly, BRCA1 deficiency has also been linked to POA and SETX deletion has been observed in ovarian cancers with a similar extent than BRCA1 deletions [72, 73].

The functional link between SETX activity and oxidative stress is not restricted to the female germline. AOA2 cells are characterized by an increased sensitivity to oxidative DNA damage insults such as H_2_O_2_, camptothecin, and mitomycin C, but not to ionizing radiation [30]. Importantly, the increase of oxidative DNA damage was associated with reduced rejoining of H_2_O_2_-induced DSB and enhanced chromosomal instability in response to H_2_O_2_. Along the same line, in Hela cells SETX loss is associated with impeded mtDNA replication and increased levels of intracellular reactive oxygen species (ROS) [74]. These events culminate in R-loop accumulation in mitochondrial DNA (mtDNA). Intriguingly, BRCA2, a SETX interacting protein, has been also implicated in mtDNA replication [74]. BRCA2 inactivation leads to R-loops accumulation in the regulatory non-coding region of mtDNA, which in turn induces diminished mtDNA replication and mtDNA deletions, typical mitochondrial dysfunctions observed in human cancers [75–79].

All together, these data indicate that defects of SETX activity impact the fidelity of DNA repair and DNA damage response signaling, induce transcription stress and lead to increased levels of DNA damage, which ultimately result to genomic rearrangements and genomic instability.

### SETX and replicative stress

It is well known that transcription may constitute a barrier for DNA replication mainly when replication and transcription machinery collides in head-on conformation on the lagging strand DNA [80, 81]. The head-on replication-transcription conflicts are particularly relevant for those genomic loci in which transcription is delayed such as fragile sites, which often contains long genes, telomers or repetitive sequences. Several lines of evidence indicate that SETX activity is instrumental to dampen persistent R-loops formation occurring during transcription replication conflicts [34, 35] (Fig. 2C). The initial observation on the role of SETX at the intercross between replication and transcription emerged by studies in yeast [35]. It has been showed that Senp1 associates with the replication forks promoting their progression across RNA pol II-transcribed genes. Senp1 mutant strains display stalled replication forks and accumulation of R-loops which in turn triggers unscheduled recombination events and genome instability. Similarly to Senp1, human SETX has been involved in dampening the detrimental effect of replication-transcription conflicts [34]. In S/G2 phase, SETX is localized into distinct nuclear foci, whose assembly is dependent on active transcription and R-loops formation. These foci increase in number upon replication stress and colocalize with the 53BP1 to sites of collision between transcription and replication. In addition to interact with components of transcription machinery, SETX has also been found to associate with the replication proteins RFC2, RFC4, and RFC5 [34]. Altogether these data suggest that in human cells SETX prevents the persistent accumulation of R-loops during transcription replication conflicts, dampening the detrimental effects of replicative stress. Another example linking SETX and replicative stress has been described in hypoxic cells [82]. Hypoxic cells are characterized by an increased level of replicative stress, likely due to decreased nucleotide availability, which can lead to R-loop accumulation [83, 84]. Ramachandran and co-authors showed that in response to hypoxia, the expression of SETX is markedly increased in a UPR dependent manner. In hypoxic cells SETX loss increases R-loop levels, leads to DNA damage and decreases DNA replication, suggesting that the hypoxia-driven induction of SETX protects cells from transcription-associated DNA damage.

### Inflammation: a possible link between SETX and tumorigenesis

**Fig. 3:**
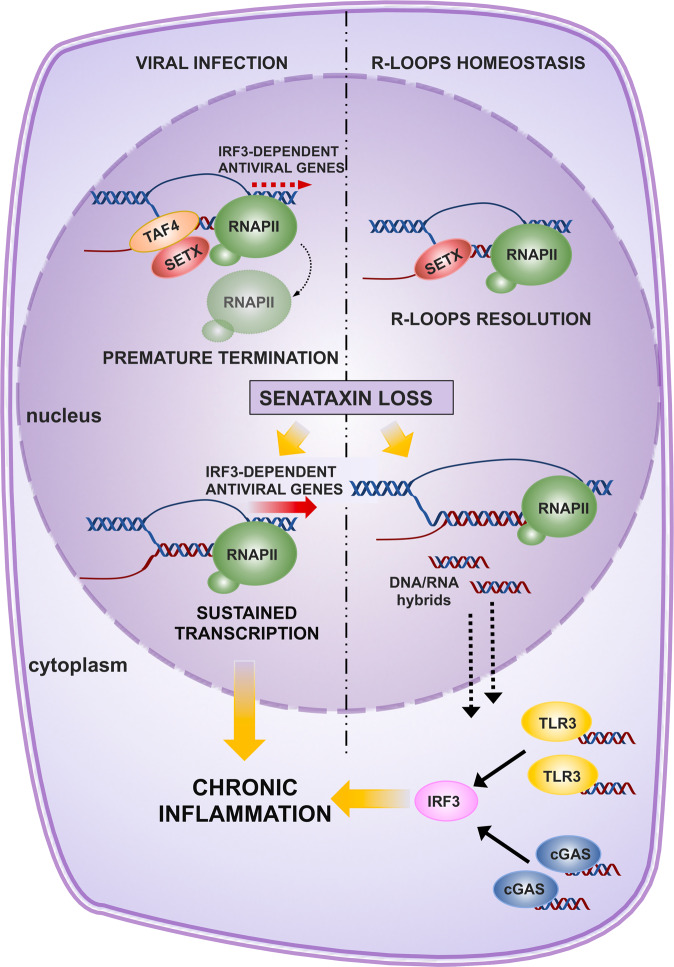
Schematic diagram of the role of SETX in inflammation. Upon viral infections (left) SETX promotes premature termination at IRF3-dependent antiviral genes. SETX loss results in increased expression of antiviral mediators in response to infection and possibly chronic inflammation. SETX-deficient cells display accumulation of cytoplasmatic DNA/RNA hybrids (right) which are products of R-loop processing. In the cytoplasm, DNA/RNA hybrids activate the immune receptors cGAS and TLR3, driving IRF3-dependent signaling and inflammation. See text for details.

Inflammation signaling represents an essential physiological cellular response of the innate immunity involved in many biological processes, such as tissue repairing and defense against pathogens. However, chronic inflammation response has been largely proved to be related to pathogenesis of several human diseases, including cancer [85–89]. In the last years, several observations have functionally linked R-loops homeostasis to the activation of the inflammation response (Fig. 3). Mutations of RNase H2 or SAMHD1, two R-loop processing enzymes, are known to trigger the activation of a robust proinflammatory response [90–93].

More recently, dysregulation of R-loops homeostasis by SETX loss has been linked with the activation of innate immune response [94]. In detail, SETX-deficient cells display accumulation of cytoplasmatic DNA/RNA hybrids which are products of R-loop processing. In the cytoplasm, DNA/RNA hybrids activate the immune receptors cGAS and TLR3, which in turn drives IRF3- dependent signaling that at high levels may induce apoptosis.

Notably, SETX and IRF3-dependent signaling are also interconnected during virus infection. SETX is indeed able to attenuate the transcription of IRF3-dependent antiviral genes during the early phase of viral infection [43]. Accordingly, SETX-deficient cells exhibit hyper-activation of the innate immune response, which ultimately results to a state of excessive inflammation [43]. The picture emerging from these data is that the R-loops resolving activity of SETX prevents the aberrant production of cytoplasmatic RNA/DNA hybrids which in turn may activate the cGAS-STING-IRF3-dependent inflammatory response. Accordingly, upon bacterial infection mouse bone-marrow-derived dendritic cells (BMDCs) derived from SETX knock-out mice displays a marked increase of inflammatory genes such as Il6 and Ifnb. Interestingly, this phenotype has not been observed in Setx^L389S+/−^ knock-in animals suggesting that gain or loss of function SETX mutations differently impact inflammatory response, leading likely to different biological outcomes [95].

In the tumor context IRF3 activation by R-loops dysregulation should represent a cellular defense mechanism to limit the propagation of genomically unstable cells harboring aberrant DNA or RNA metabolism. Accordingly, in cancerous and immune cells cGAS-STING signaling has been linked to induction of cell death, cell senescence, and with the activation of antitumor immunity. However, the precise role of cGAS-STING signaling on cancer occurrence and progression is controversial. Indeed, several evidence indicates that this pathway may favor tumor metastasis and facilitate cancer progression [96, 97]. It is likely that the impact of SETX-cGAS axis on tumor biology is context- and stage-dependent and additional studies are required to clarify this issue.

## SETX mutations: beyond neurological disorders

**Fig. 4:**
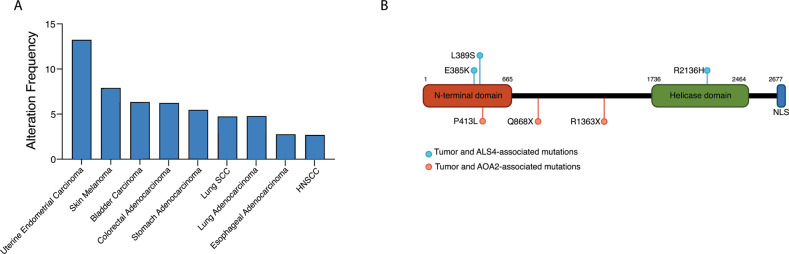
*SETX* mutations in human tumors. **A)** Percentage of mutation and deep deletion in the indicated human tumors based on cBIOPortal for Cancer Genomics (http://www.cbioportal.org/public-portal). **B)** Location of SETX mutations common between human tumors and ALS4 (blue) and AOA2 (red).

In addition to neurological diseases, SETX dysregulation has been also observed in human tumors and several evidence pointed towards a tumor suppressive role of this helicase (Fig. 4A). SETX has been identified in a screen for tumor suppressor genes and its expression is markedly downregulated in diverse human cancer types. For instances, SETX expression is downregulated in ovary carcinoma, lymphomas, primary Basal Cell Carcinoma (BCC) and Squamous Cell Carcinoma (cSCC) arising from different anatomical sites such as skin, cervical and lung [45, 98]. In addition to dysregulated expression, human tumors are also characterized by mutations in *SETX* gene and a tumor suppressive screening identified SETX as a mutated gene in breast cancer [99]. Tumor-associated SETX mutations are present throughout the entire sequence and include missense, truncating and splice site mutations (cBIOPortal for Cancer Genomics; http://www.cbioportal.org/public-portal). Some SETX residues are more prone to undergo mutations. For instances, R1623X mutations have been reported in in 4 cases of Uterine Endometrioid Carcinoma, 2 cases of stomach carcinoma and 2 cases of colon adenocarcinoma. Tumors with the highest rate of SETX mutations are uterine endometrial carcinoma (13,23%), skin melanoma (7,6%) and colon adenocarcinoma (6,23%). Remarkably, cutaneous SCC patients harboring SETX mutation showed a decrease of the overall survival compared to wild-type counterpart. Three AOA2-related *SETX* mutations, Q868X, R1363X, and P413L have been reported in Uterine Carcinoma, colon carcinoma and skin melanoma, respectively, suggesting that loss of SETX function might be beneficial for tumor development and progression (Fig. 4B). However, this view is likely to be too simplistic and it is reasonable that the impact of SETX dysregulation on tumorigenesis might be context- and stage-dependent. To further complicate this scenario, AOA2 patients do not show an increase of cancer susceptibility suggesting that other RNA/DNA helicases could compensate for SETX loss. Furthermore, a study analyzing the non-neurological clinical features of 32 ALS4 patients harboring the L389S or E385K gain of function mutations of SETX revealed that five ALS4 patients have benign neoplasia of the colon (colonic polyps) and two display malignant lesions (adenocarcinoma of the colon) [100]. Remarkably, the early onset of colonic polyps and colon adenocarcinoma (one individual with colon adenocarcinoma is 35‐year‐old) raises the possibility that *SETX* gain of function mutations might be an early event accelerating tumor onset.

## Concluding remarks and perspectives

Over the past years, multiple evidence have clearly established the critical role of the R-loops resolving enzyme SETX in different physiological and pathological processes. The most compelling evidence that SETX dysregulation affects human health is the finding that gain or loss of function mutations of *SETX* gene underlying the pathogenesis of two distinct neurological disorders. In addition to neurological diseases, SETX mutations have been also reported in human tumors and several observations pointed towards a tumor suppressor function. These observations include: (i) SETX gene is frequently mutated or its expression is downmodulated in different malignancies; (ii) R-loops are generally considered oncogenic events since their unscheduled formation affects genome integrity; (iii) SETX controls DNA damage response and DNA repair mechanisms whose dysregulation is frequently observed in many, if not all, tumors; (iv) SETX physically interacts with the tumor suppressor gene BRCA1 to promote DNA repair at the transcribed loci; (v) SETX together with the exonuclease SAN1 is able to induce the repair of ICLs whose defects are associated with the cancer susceptibly syndrome Fanconi anemia (FA); (vi) SETX attenuates the replication stress, which in the tumor context is an early event triggered by the activity of many oncogenes; (vii) SETX activity is involved in inflammatory response which is often deregulated in many human tumors.

Although these observations suggest a potential tumor suppressive role of SETX, the genetic and molecular evidence of the direct involvement of SETX defects in tumor onset and progression has not been provided so far. The lack of the cancer susceptibility phenotype in AOA2 patients as well as the absence of spontaneous tumorigenesis in SETX knock-out mice further complicates our comprehension of the potential impact SETX on tumor development. Furthermore, it is not clear which specific SETX functions underlie its potential tumor suppressive role. Analysis of AOA2 and ALS4 cells has clearly indicated that the unscheduled turnover of R-loops is a central hallmark of those neurological disorders. However, as discussed above, alterations of R-loops homeostasis by SETX dysregulation impinge on many processes, each of them potentially contributing to the development of neurological disorders as well as tumor development. Furthermore, it is possible that not only loss of function but also gain of function alterations might drive tumor development, similarly to what is observed in the neurological context. While AOA2 patients do not show an increase of cancer susceptibility, a cohort of ALS4 patients harboring SETX L389S mutation show the presence of benign and malignant intestinal lesions linking colon carcinoma development with SETX gain of function mutations. Although this phenotype has been reported in a single study and the molecular pathways underlying this phenotype are missing, we can argue that the biological outcome exerted by an efficient SETX enzyme, as much as one that removes R-loops inefficiently, could be instrumental for tumor development. For instances, in contrast to AOA2, ALS4 mutations affect the TGF-beta pathway which is commonly dysregulated in human colon adenocarcinoma. ALS4 mutations do not interfere with SETX sumoylation, which is required of its interaction with the RNA exosome complex at sites of replication-transcription conflicts [27, 44]. It is possible that an increase in the SETX activity is beneficial at the early stage of tumor growth when cells need to face the detrimental effect of oncogene-induced replicative stress [101–105]. In support of this, hypoxia increases SETX expression and, by doing this, limits replication stress. Alternatively, the increase of the R-loop resolving activity of SETX might dampen the pro-apoptotic effect gGAS-STING pathways activated by the formation of cytoplasmatic DNA/RNA hybrids, limiting thus the removal of genetically unstable cells.

Another consideration emerging from these observations is that SETX levels need to be tightly regulated since slight alterations of its expression or activity may induce dramatic effect on cell homeostasis. Our understanding of the mechanisms regulating SETX expression is still limited. A recent report demonstrated that similarly to yeast Senp1, SETX protein levels are regulated by the ubiquitin proteasome system [106], a regulatory mechanism critically involved in tumor progression and evolution [107–114]. In detail, SETX proteostasis is regulated by the crosstalk between the E3 ubiquitin ligase KEAP1 and the deubiquitylation enzyme USP11 [115]. Intriguingly, KEAP1-USP11 defective tumor cells exhibit BRCAness phenotype, i.e., alteration of R-loops homeostasis, DNA damage, and higher sensitivity to PARP inhibitors [116, 117]. Therefore, SETX might represent a therapeutically actionable target to modulate the cytotoxic response of those tumor types carrying aberrant USP11-KEAP1-NRF2 pathway [118–120]. Therefore, a deeper comprehension of the circuits controlling SETX expression might be relevant to unveil novel therapeutically actionable routes in neurological disorders as well as human cancers.

SETX is ubiquitously expressed. Nevertheless, SETX mutations affect only limited types of human tissues leading mainly to neurological disorders and defects in germline homeostasis. It is possible that these tissues are more sensitive to R-loop unscheduled turnover due to the type of transcribed genes or to the lack of an efficient compensatory mechanism. The same scenario might take place during tumor onset and only specific tissues might be sensitive to SETX dysregulation.

In conclusion, these observations will certainly fuel future research aimed to formally clarify the impact of SETX on tumor evolution and enlighten the ambiguous boundary between the physiological and pathological outcome of SETX-driven R-loops resolution. The analysis of the functional consequences of SETX loss in mice models of cancer research will help our understanding of how and whether this important DNA/RNA helicase may impact tumor development and progression.

## Data Availability

The data that support the findings of this study are openly available in a public repository.
